# A modified chain-based sponge dressing controls junctional hemorrhage in the tactical combat casualty care simulation of pigs

**DOI:** 10.1186/s13049-023-01133-6

**Published:** 2023-11-09

**Authors:** Weihang Wu, Wangwu Liu, Nan Lin, Hu Zhao, Jin Yang, Zhi Ye, Weijin Yang, Yu Wang, Yongchao Fang

**Affiliations:** Department of General Surgery, Fuzong Clinical Medical College of Fujian Medical University, 900TH Hospital of Joint Logistics Support Force, PLA, Fuzhou, Fujian 350025 China

**Keywords:** Modified chain-based sponge dressing, Polyvinyl alcohol, Bullet penetrating wound, The junction, First aid of Hemorrhage

## Abstract

**Background:**

Hemorrhage has always been the focus of battlefield and pre-hospitalization treatment. With the increasing fatality rates associated with junctional bleeding, treatment of bleeding at junctional sites has gradually gained attention in battlefield trauma emergency care. We designed a modified chain-based sponge dressing with a medical polyvinyl alcohol sponge that can be used to treat junctional hemorrhage and tested its hemostatic efficacy and biocompatibility.

**Methods:**

Twenty adult Bama miniature pigs were randomly divided into the modified chain-based sponge dressing (MCSD) and standard gauze (SG) groups. The right femoral artery of the pigs was shot at after anesthesia. The Bama miniature pigs were moved to the safety zone immediately to assess the condition according to the MARCH strategy, which evaluates massive hemorrhaging, airway obstruction, respiratory status, circulatory status, head injury & hypothermia. Hemoglobin and coagulation status were checked during the experiment.Among the pigs in which the inguinal hemorrhagic model based on bullet penetrating wounds was successfully established, those in the MCSD group received a disinfected MCSD for hemostasis, while those in the SG group received standard gauze in an imbricate manner to pack the bullet exit and entrance wounds to stop bleeding until the wound was filled, followed by compression for 3 min at sufficient pressure. CT scanning, transmission electron microscopy, and HE staining were conducted after experiment.

**Results:**

The MCSD group showed lower hemostasis time and blood loss than the gauze group. The MCSD group also showed a higher success rate of treatment,more stable vital signs and hemoglobin level. The CT scanning results showed tighter packing without large gaps in the MCSD group. The histopathological assessments and the transmission electron microscopy and HE staining findings indicated good biocompatibility of the polyvinyl alcohol sponge.

**Conclusion:**

The MCSD met the battlefield’s requirements of speedy hemostasis and biosafety for junctional hemorrhage in Bama miniature pigs. Moreover, in comparison with the conventional approach for hemostasis, it showed more stable performance for deep wound hemostasis. These findings provide the theoretical and experimental basis for the application of MCSD in the treatment of hemorrhage in the battlefield in the future.

## Background

Between 1990 and 2000, approximately 36,000 people died in localized war conflicts every year [1]. Blast-related injuries and bullet wounds are the most common types of injuries on the battlefield. More than 70% of the wounded individuals die within 1 h of being injured, with the main causes of death being central nervous system injury (31%), hypovolemia after massive hemorrhage (30%), burns (21%), and combined injuries (11%) [2]. Research suggests that hemorrhage-related death can be prevented by emergency hemostasis [3]. In conjunction with the decline in mortality caused by injuries of the extremities due to the use of limb tourniquets, the proportion of deaths from hemorrhage at the junctions of the trunk and extremities, such as the groin, neck, armpits, and buttocks, has gradually increased. In a retrospective analysis of 4596 war-wound-related deaths, the mortality rate attributable to junctional hemorrhage (19.2%) surpassed that attributable to injuries to the extremities (13.5%) and has shown a continuing upward trend [3, 4]. Thus, quick and effective self-rescue and mutual rescue for massive junctional bleeding has become the focus of battlefield rescue. However, application of the recommended hemostatic materials for junctional bleeding presents disadvantages such as the inability to stop bleeding from deep injuries, high difficulty in operation, inconvenient portability, and residual material after removal.

To address the problems associated with junctional bleeding, we designed a chain-based sponge dressing (CSD) based on medical-grade polyvinyl alcohol (PVA) sponge [5]. The sponge in the wound achieved deep hemostasis by sucking blood, similar to XStat. The XStat device is a hemostatic system composed of multiple mini-sponges, but the use of independent mini-sponges may lead to residual material in the wound. By connecting the sponges using non-absorbable surgical sutures, our CSD could reduce the possibility of residual material in the wound while ensuring that the dressing fell out after hemostasis. We had previously verified the efficacy of CSD in an inguinal bullet penetrating wound hemorrhagic model [6]. However, in the previous experiments, the PVA sponges expanded when they encountered blood on the surface of the wound due to the short injection head of the original chain hemostatic device, reducing the effectiveness of the CSD device for deep wound hemostasis. To address this limitation, the device was further modified, and we have verified the hemostasis effect and safety of the modified chain hemostatic dressing (MCSD) by simulating tactical combat casualty care for junctional bleeding in this study.

## Materials and methods

### Materials

**Fig. 1:**
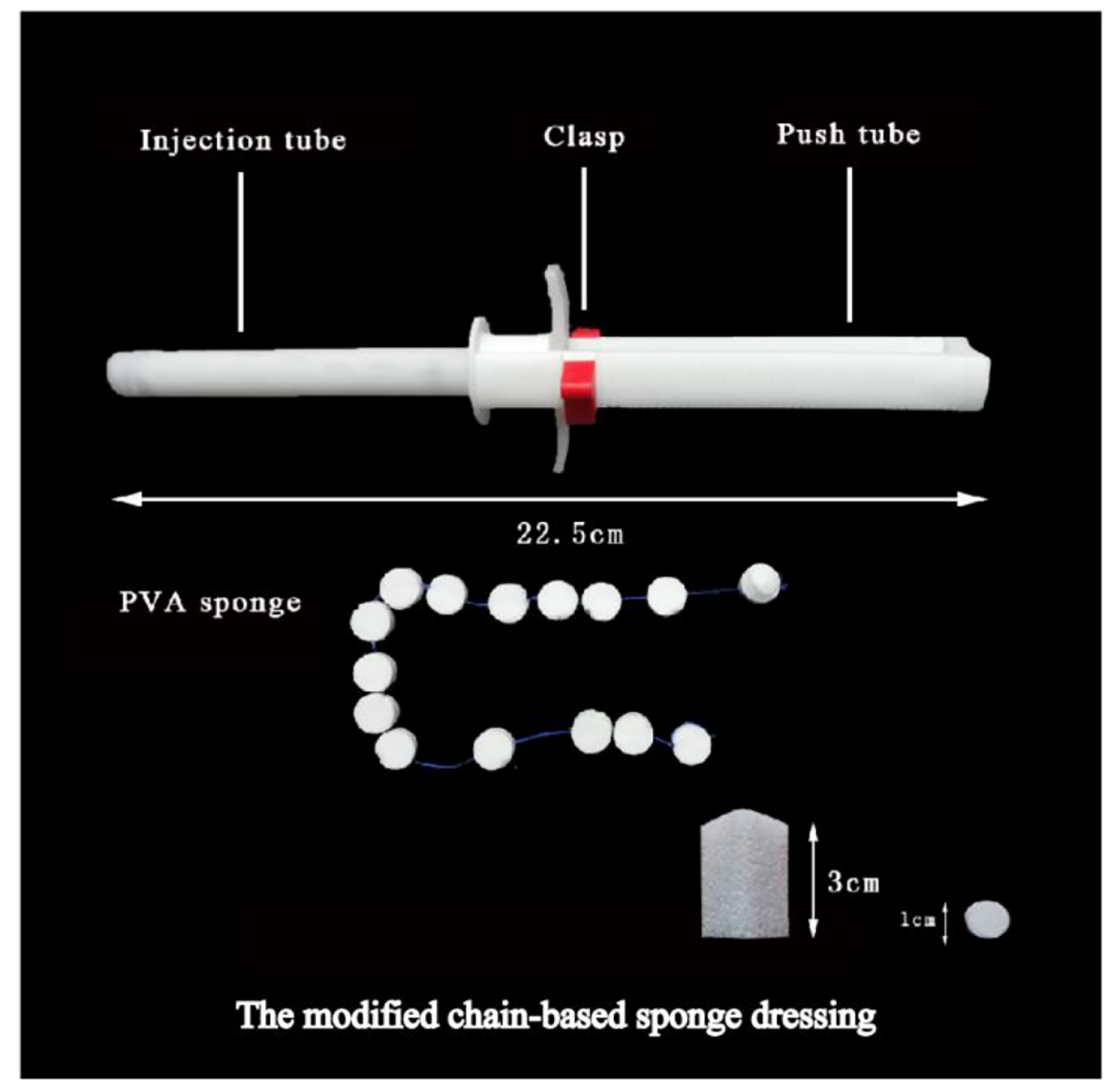
Schematic diagram of the modified chain-based sponge dressing

The PVA sponge was developed by Fuzhou University, Fujian province, China. It was cut into a triangular prism with a bottom side length of 2.30 × 2.30 × 2.30 cm and a height of 3.0 cm, and then compressed into a cylinder with diameter and height of 1.0 cm and 0.7 cm, respectively. Fifteen compressed PVA sponges were connected in series with non-absorbable surgical sutures and filled in a disposable polypropylene injection tube. The MCSD device was mainly composed of the injection tube (length, approximately 10 cm) and the push tube (length, approximately 12.5 cm), and the weight of a single MCSD was about 15 g. PVA sponges were stored in the injection tube in sequence. For using the MCSD, the injection tube was inserted into the wound, the red safety clasp was removed, the beam was pulled out, and the injection tube was driven backward, pushing the sponges backward into the wound (Fig. 1).

### Animals

Thirty adult Bama miniature pigs (15 males, 15 females; age, 6 months; weight, 20–30 kg) were purchased from Hubei Aofei Biotechnology Co, Ltd. All animals were raised normally. The animals were fasted for 8 to 12 h and did not receive water for 4 h on the day of the experiment. Before experiment, Sutai 10 mg/kg, luminin 2 mg/kg, and atropine 0.06 mg/kg were injected into the gluteus maximus muscle to induce anesthesia. Sutai 2.5 mg/kg and luminin 1 mg/kg were injected intravenously to maintain anesthesia. The left femoral artery was exposed and a 6 F catheter was placed in the femoral artery to monitor mean arterial pressure (MAP). The right internal jugular vein was exposed, and a central venous catheter was placed for fluid resuscitation. This experimental protocol was approved by the Ethics Committee for Drug Clinical Trials of The 900th Hospital of Joint Logistics Support Force, PLA (IEC-2021-058).

### Simulation of junction bleeding and tactical combat casualty care

Twenty adult Bama miniature pigs (males, 10; females, 10) were randomly divided into MCSD and standard gauze (SG) groups. At 30 min after induction of anesthesia and arteriovenous catheterization, the Bama miniature pig was fixed in a standing position on a special bracket with the left inguinal area facing straight forward. The body surface projection of the left femoral artery was explored and labeled by a senior ultrasound chief physician using a portable color Doppler ultrasound instrument.

An expert sniper with at least 5 years of shooting experience armed with a CS/LR4A high-precision sniper rifle with 7.62*51 mm bullets (initial velocity, about 800 m/s) shot the body surface marking of the femoral artery in the left inguinal area from a distance of 50 m. The timing was started after the shot, and natural bleeding was allowed for 30 s. The Bama miniature pigs were then moved to the safety zone immediately to assess the condition according to the MARCH strategy. First, the massive hemorrhaging status (M) of the open wound was evaluated. Among the pigs in whom the inguinal bullet penetrating wound hemorrhagic model was successfully established, those in the MCSD group received a disinfected MCSD for hemostasis. In the SG group, standard gauze was used to pack the bullet exit and entrance wounds in an imbricate manner to stop bleeding until the wound was filled and pressed for 3 min at sufficient pressure. Evaluation for active bleeding was performed after filling both entrance and exit wounds. Continued active bleeding indicated failure of hemostasis. During hemostasis, gauze and cotton pads were used to collect the outflowing blood. Subsequently, the airway obstruction (A), respiratory findings such as lip color, blood oxygen saturation, and respiratory frequency (R), circulatory findings such as carotid pulse, MAP, heart rate, and shock index (C), and head injury & hypothermia (H) were evaluated. Vital signs such as shock index, central venous pressure (CVP), and MAP, and blood test results such as the hemoglobin level and platelet count were recorded at 0,0.5, 30, 60, and 120 min.

At 2 h after the experiment, one Bama miniature pig each from the MCSD and SG groups was randomly selected for pelvic CT scanning. All animals were observed for 3 h or until death, after which the MCSD or gauze was removed to observe whether hemorrhage occurred again. After the hemostatic materials were taken out, the wound in the groin area and the injuries of the femoral artery and femoral vein were examined. The broken end of the femoral artery and surrounding muscle tissue were obtained for HE staining. The PVA sponges used for compression, expansion, and blood suction and the muscle tissue around the femoral artery were harvested for transmission electron microscopy (TEM). To minimize the effects of excess fluid resuscitation on the experimental data, rehydration was performed using only LR solution at a rate of 10 mL/min to maintain MAP. Death was determined when the MAP reduced below 20 mmHg and the heart rate was 0, and the time of death was recorded. After the experiment, all surviving animals were euthanized with 10% potassium chloride injection at 0.5 mL/kg.

### Statistical processing

The statistical software PASS11.0 was used to estimate the sample size before the experiment. SPSS 26.0 (IBM Corp., Armonk, NY, USA) was used for statistical analyses. The Shapiro-Wilk method was used to test the normality of the data for vital signs, laboratory results, bleeding volume, hemostasis, and extraction time. Measurement data that showed a normal or approximately normal distribution were expressed as mean ± standard deviation (x ± s), and measurement data that did not show a normal distribution were expressed as median (interquartile range). The independent-sample *t* test was used to compare the baseline data, bleeding volume before and after hemostasis, hemostasis time, and removal time between the two groups. GraphPad Prism 9.0 was used to perform two-way ANOVA with the multi-point repeated measurement data of vital signs and laboratory results of the two groups, and p < 0.05 was considered to indicate statistically significant differences.

## Results

Among the 20 Bama miniature pigs selected for the experiment, one died due to anesthesia intolerance before modeling, and three others did not show femoral artery injuries during wound exploration after the experiment; these four animals were excluded from the subsequent analyses. Thus, the sample size was n = 10 in the MCSD group and n = 6 in SG group. The MCSD and SG groups showed no significant differences in baseline data (Table 1).

**Table 1:** Baseline data of the Bama miniature pigs before establishment of the inguinal bullet penetrating wound model

Parameter	MCSD group (n = 10)	SG group (n = 6)	*P-*value
Weight (kg)	28.13 ± 5.35	26.93 ± 7.59	0.716
Temperature (°C) Heart rate (beats/min)	37.75 ± 0.47 90.60 ± 7.75	37.53 ± 0.75 89.67 ± 7.55	0.485 0.817
Mean arterial pressure (mmHg) Respiratory rate (breaths/min) Oxygen saturation (%)	96.40 ± 20.9 39.10 ± 5.91 96.60 ± 1.71	99.33 ± 17.08 37.83 ± 9.41 96.00 ± 2.19	0.777 0.744 0.550
Central venous pressure (cmH_2_O)	6.20 ± 2.22	7.42 ± 2.88	0.357
Hemoglobin (g/L)	129.00 ± 12.73	133.00 ± 7.82	0.501
Blood platelet count (1000/µL)	446.50 ± 74.20	436.20 ± 32.61	0.781
Fibrinogen (mg/L) PT (s)	1.69 ± 0.39 10.68 ± 1.08	1.69 ± 0.51 11.86 ± 1.32	0.985 0.094
APTT (s)	24.31 ± 4.42	30.80 ± 12.33	0.227

PT, prothrombin time; APTT, activated partial thromboplastin time.

### Hemostatic effect

**Fig. 2:**
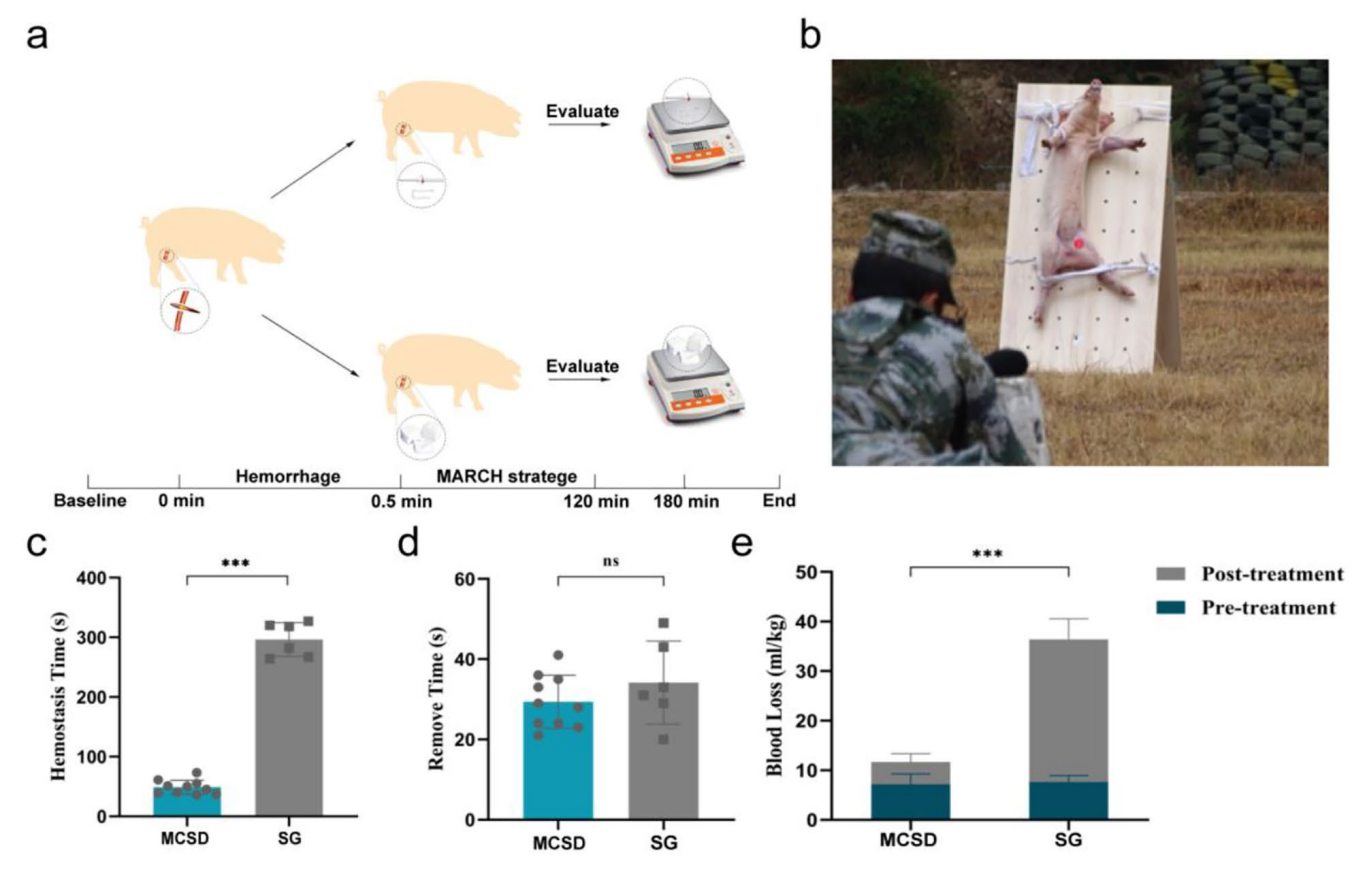
The hemostatic effect of MCSD on inguinal bullet penetrating wounds. **(a)** Schematic diagram of the tactical combat casualty care simulation of junctional bleeding: after 30 s of free bleeding, the two groups received the MCSD or gauze to stop the bleeding, and vital signs were recorded according to the MARCH strategy within 2 h. After 3 h, the hemostatic materials were taken out and the wound was evaluated. **(b)** The Bama miniature pigs were fixed at 50 m and shot. **(c)** The hemostasis time in the MCSD and SG groups. **(d)** The amount of blood loss in the MCSD and SG groups. **(e)** The removal time of hemostatic material in the MCSD and SG groups

The treatment process is shown in Fig. 2a. The hemostasis time in the MCSD group (48.70 ± 11.87 s) was significantly shorter than that in the SG group (296.33 ± 28.57 s; p < 0.001; Fig. 2b). The blood loss before treatment in the MCSD and SG groups was 7.19 ± 0.66 mL/kg and 7.64 ± 0.54 mL/kg, respectively, with no significant difference between the groups (p > 0.05). The post-treatment blood loss in the MCSD group (4.47 ± 1.68 mL/kg) was less than that in the SG group (28.76 ± 4.14 mL/kg; p < 0.001). The total blood loss was 11.67 ± 3.52 mL/kg in the MCSD group and 36.40 ± 5.23 mL/kg in the SG group, with a statistically significant difference between the groups (p < 0.001; Fig. 2c). The two groups showed no significant difference in the time of material removal (p > 0.05; Fig. 2d).

**Fig. 3:**
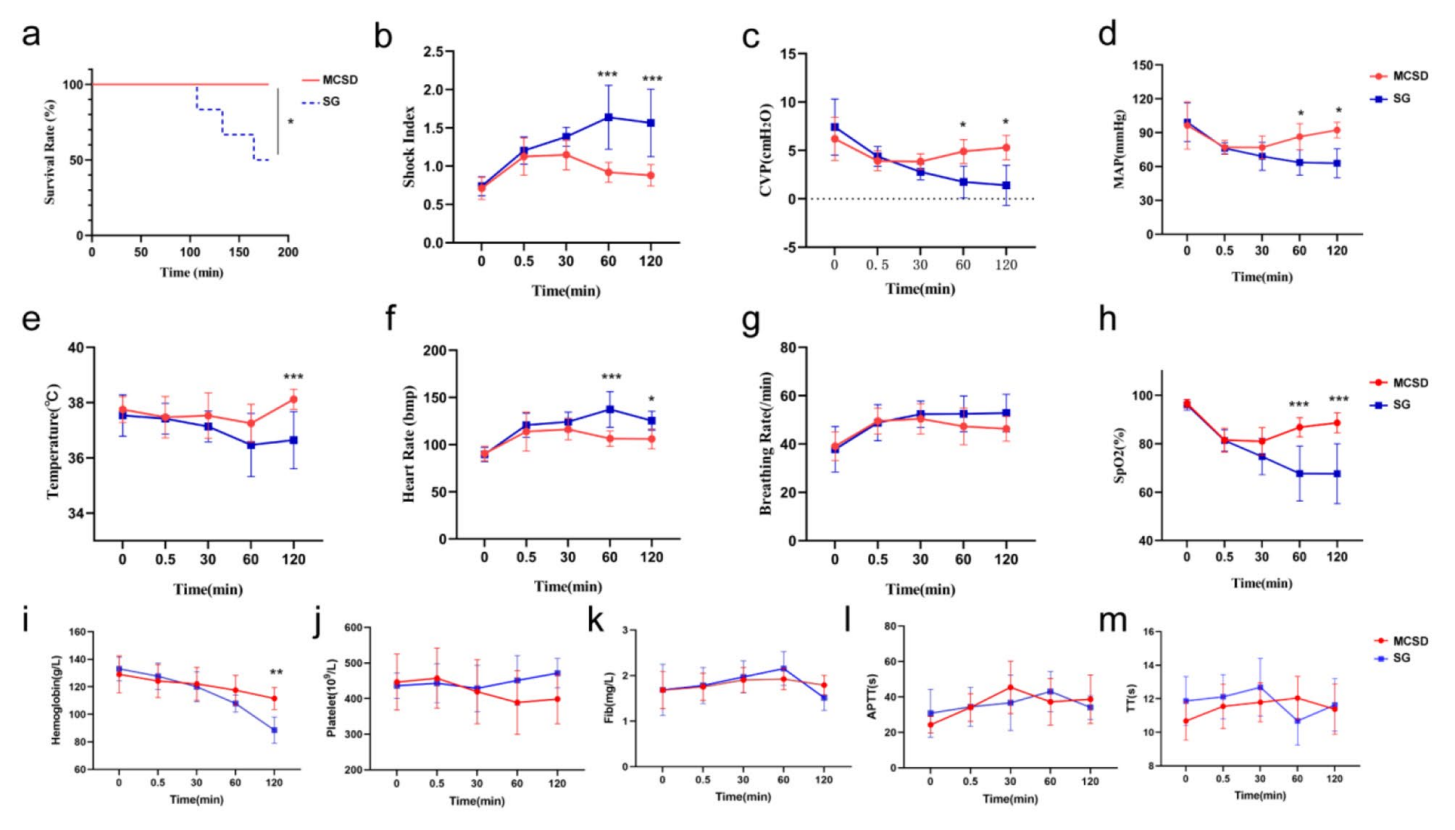
Trends of vital signs and laboratory results in the Bama miniature pigs. **(a)** Survival rate; **(b)** shock index; **(c)** central venous pressure; **(d)** mean arterial pressure ; **(e)** temperature; **(f)** heart rate; **(g)** breathing rate; **(h)** oxygen saturation; **(i)** hemoglobin level; **(j)** platelet count; **k.** fibrinogen level; **l.** activated partial thromboplastin time; **m.** thrombin time (*: p < 0.05; * *: p *<* 0.01; ***: p < 0.001)

After the ballistic trajectories were filled with the PVA sponge, no blood continued to ooze out of the 10 Bama miniature pigs in the MCSD group. Thus, the success rate of hemostasis in the MCSD group was 100% (10/10), and all animals in the MCSD group survived to 180 min. In the SG group, three Bama miniature pigs continued to show bleeding after hemostasis, and the success rate of hemostasis was 50% (3/6). The three pigs without successful hemostasis died at 107 min, 133 min, and 165 min. The cause of death was hemorrhagic shock in all three pigs. The rest of the animals in the SG group survived to 180 min, with a significant difference in the survival rate between the two groups (p < 0.05; Fig. 3a).

### Changes in vital signs

#### Shock index

An analysis of vital signs data at different time points is shown in Fig. 3. The shock index increased after hemorrhage in both groups, with no significant difference between the two groups at 30 s and 30 min (1.13 ± 0.24 in the MCSD group vs. 1.21 ± 0.18 in the SG group at 30 s, p > 0.05; 1.15 ± 0.20 in the MCSD group vs. 1.38 ± 1.22 in the SG group at 30 min, p > 0.05), and statistically significant differences at 60 and 120 min (0.92 ± 0.13 vs. 1.63 ± 0.42 at 60 min, p < 0.001; 0.88 ± 0.14 vs. 1.57 ± 0.44 at 120 min, p < 0.001; Fig. 3b).

#### Central venous pressure

CVP decreased in both groups after bleeding, with no significant difference between the two groups at 30 s and 30 min (3.93 ± 1.02 cmH_2_O in the MCSD group vs. 4.38 ± 1.03 cmH_2_O in the SG group at 30 s, p > 0.05; 3.85 ± 0.80 in the MCSD group vs. 2.78 ± 0.80 cmH_2_O in the SG group at 30 min, p > 0.05), and a statistically significant difference in CVP at 60 and 120 min (4.90 ± 1.24 vs. 1.75 ± 1.63 cmH_2_O at 60 min, p < 0.05; 5.05 ± 1.30 vs. 1.30 ± 1.70 cmH_2_O in 120 min; p < 0.05; Fig. 3c).

#### Mean arterial pressure

The curve of the MAP is shown in Fig. 3d. Due to massive bleeding within 30 s, the MAP of Bama miniature pigs decreased in the MCSD and SG groups. After applying MCSD and SG respectively to stop bleeding, the MAP in the MCSD group did not further decrease due to successful hemostasis. However, the blood loss in the SG group was significantly greater than that in the MCSD group due to the longer hemostasis time and the failure of hemostasis in three Bama miniature pigs in the SG group.

#### Temperature

The body temperature of Bama miniature pigs in the MCSD and SG groups decreased after bleeding, with no significant intergroup differences at 30 s, 30 min, and 60 min. However, the two groups showed a statistically significant difference in body temperature at 120 min (38.12 °C ± 0.36 °C in the MCSD group vs. 36.64 °C ± 1.03 °C in the SG group; p < 0.001; Fig. 3e).

#### Heart rate

The penetrating bullet wounds in the groin caused substantial blood loss, resulting in changes in the heart rate of the Bama miniature pigs. The two groups showed no significant differences in heart rate at 30 s and 30 min after bleeding (113.90 ± 20.58 beats/min vs. 120.67 ± 12.79 beats/min at 30 s in the MCSD and SG groups, p > 0.05; 116.30 ± 11.23 beats/min vs. 124.17 ± 10.32 beats/min at 30 min, p > 0.05), but showed significant differences in heart rate at 60 and 120 min, respectively (106.40 ± 8.14 beats/min vs. 137.33 ± 18.99 beats/min at 60 min, p < 0.001; 106.10 ± 10.55 beats/min vs. 125.40 ± 10.07 beats/min at 120 min, p < 0.05; Fig. 3f).

#### Respiratory rate

After bleeding, the respiratory rate of both groups was accelerated to different degrees, but showed no significant differences between the two groups (49.50 ± 5.36 breaths/min vs. 48.83 ± 7.47 times/min at 30 s in the MCSD and SG groups, p > 0.05; 50.40 ± 6.22 breaths/min vs. 52.33 ± 5.47 breaths/min at 30 min, p > 0.05; 47.30 ± 7.57 breaths/min vs. 52.50 ± 7.34 breaths/min at 60 min, p > 0.05; 46.30 ± 5.14 breaths/min vs. 52.80 ± 7.79 breaths/min at 120 min, p > 0.05).

#### Blood oxygen saturation

Blood oxygen saturation decreased in both groups after bleeding. It showed no significant difference between the two groups at 30 s and 30 min (81.50% ± 4.88% vs. 81.33% ± 4.46% at 30 s, p > 0.05; 81.00% ± 5.71% vs. 74.67% ± 7.53% at 30 min, p > 0.05), but differed significantly between the groups at 60 and 120 min (86.80% ± 3.93% vs. 67.67% ± 11.38% at 60 min, p < 0.001; 88.60% ± 4.14% vs. 67.60% ± 12.36% at 120 min, p < 0.001; Fig. 3h).

### Assessment of bleeding-related laboratory results

#### Hemoglobin level

Hemoglobin levels decreased in both groups after bleeding, with no significant difference between the two groups at 30 s, 30 min, and 60 min (124.2 ± 11.38 g/L vs. 127.7 ± 8.73 g/L at 30 s, p > 0.05; 122.1 ± 11.39 g/L vs. 120.0 ± 9.88 g/L at 30 min, p > 0.05; 117.5 ± 10.29 g/L vs. 107.8 ± 5.67 g/L at 60 min, p > 0.05), while the values at 120 min were significantly different between the two groups (111.5 ± 7.58 g/L vs. 88.5 ± 8.71 g/L, p < 0.01; Fig. 3i).

#### Coagulation indicators

The coagulation indicators, i.e.,blood platelet,fibrinogen level, activated partial thromboplastin time, and thrombin time, in Bama miniature pigs did not differ significantly between the groups in measurements obtained at 30 s, 30 min, 60 min, and 120 min (Fig. 3j-m).

### Evaluation of local application reactions

**Fig. 4:**
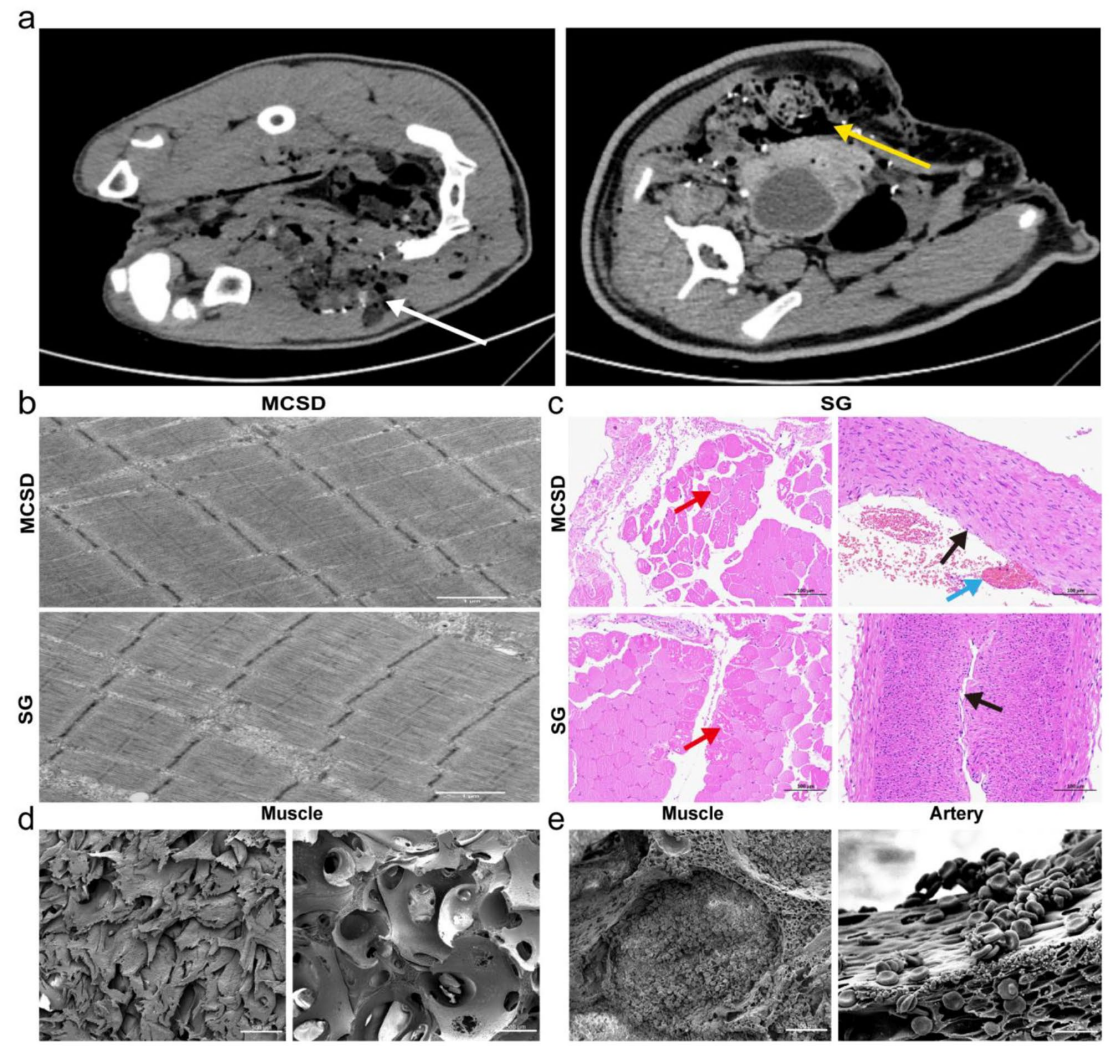
Evaluation of local application reactions. **a.** CT scan results of the Bama miniature pig. **b.** Transmission electron microscopic images of muscle tissue (scale bar = 1 μm). **c.** HE staining of the femoral artery stump and muscle (scale bar = 100 μm). **d.** The dense porous structure of the PVA sponge in the compressed state (left); the specific surface area of the PVA sponge increased significantly after water absorption (right; scale bar = 500 μm). **e**. A large number of red blood cells adhered to the PVA sponge after absorption of blood (left: scale bar = 100 μm, right: scale bar = 20 μm)

The CT scan results showed that the bullet penetrated the groin area to form a penetrating injury. Gray density shadows formed by the PVA sponge and gauze could be seen in the penetrating wound formed by the bullet. The MCSD group showed tighter packing without a large gap (white arrow in Fig. 4a), while the SG group showed a larger gap residue (yellow arrows in Fig. 4a).

The TEM images of muscle tissue indicated that the muscle filaments in the MCSD and SG groups were well arranged, with some swollen mitochondria and no obvious necrosis of myocytes (Fig. 4b). HE staining of the femoral artery stump after the application of MCSD and SG showed the absence of arterial endothelial cells (black arrows in Fig. 4c), some swollen medial cells, and red blood cells aggregated in the arterial wall (blue arrows). A small amount of myocyte necrosis was observed in the muscle tissue around the femoral artery, the nucleus was hyperstained or dissolved, the cytoplasm was eosinophilic, and the rest of the tissue was not necrotic (red arrow in Fig. 4c). Overall, the application of MCSD did not lead to femoral artery necrosis or significant necrosis of muscle tissue.

## Discussion

Hemorrhage from battlefield trauma is a vital cause of early death and the leading cause of preventable death. At present, the most commonly used animal models in trauma hemostasis experiments are pig femoral artery or carotid artery injury hemorrhage models [7, 8], and injury hemorrhage models caused by military weapons are rare. However, gunshot wounds tend to have a cavity area larger than the outlet, rather than being linear, and such complex deep wounds with massive bleeding are often difficult to stop [9, 10]. To address these limitations of existing models, we had successfully established the inguinal bullet wound hemorrhagic model to test the hemostatic effect of the CSD in our previous study [6].

In this study, we further adjusted the method for establishing the groin bullet wound hemorrhagic model by accounting for the guidelines of tactical combat injury treatment. First, we calculated the appropriate sample size through the statistical software and expanded the sample in comparison with the previous study. Second, to simulate the limited first aid time in an actual battlefield, the treatment was started 30 s after the successful establishment. Many other bleeding models also use 30 s as the initial time point [11, 12]. The MCSD and SG groups showed no significant differences in the changes of vital signs within 30 s. Wounds caused by bullets are highly uncontrollable, and free bleeding period of 30 s before hemostasis could not only simulate the battlefield treatment environment, but also further ensure the uniformity of blood loss between the two groups. Thirdly, a clear positive correlation has been observed between the mortality of those who are hit and the duration of first aid [13]. Therefore, we developed a timely care and evaluation protocol based on the “MARCH” strategy. Fourth, to simulate the longer shooting distance in the battlefield, we adjusted the shooting distance to 50 m and used a high-precision sniper rifle for shooting. A color Doppler ultrasound-based marking was also used to improve the accuracy. In addition, we excluded Bama miniature pigs in which the femoral artery was not injured, and we did not attempt shooting again to avoid influencing the data results. Lastly, the hemostatic methods in this experiment showed several differences: (1) The TCCC guidelines recommend that the gauze should be pressurized for at least 3 min to stop bleeding. To simulate the compressive effect of PVA sponge in deep tissues, we maintained an additional 20 kPa pressure while the gauze was packed into the wound [14]. (2) Since the hemostatic material will pull the tissue and induce bleeding if taken out again for observation, we only evaluated the success of hemostasis once in this experiment. Continued bleeding even after the PVA sponge or gauze filled the trajectory was considered to indicate hemostasis failure.

Penetrating wounds and blind wounds caused by bullets or explosive fragments often have narrow entrances and irregular shapes, making it difficult for gauze and dressings to fill wounds and effectively stop bleeding [15]. The CoTCCC recommends three hemostatic devices for the treatment of junctional bleeding: Combat Ready Clamp (CRoC), Attached Emergency Treatment Tool (JETT), and SAM Attached Tourniquet (SJT) and XStat. However, CRoC is bulky and heavy and takes a long time to assemble [16]. On the other hand, both SJT and JETT show low success rates for hemostasis, which requires training to improve [17]. The XStat™ device (RevMedx, Inc.) is a new type of hemostatic device with 92 hemostatic sponges containing chitosan. These sponges expand rapidly after being pushed into the wound, filling the deep and incompressible wound to stop the bleeding [18]. However, XStat™ is composed of many individual micro-sponges, necessitating more time to remove these sponges from the wound. In some cases, complete removal of the sponges necessitated wound enlargement, which further aggravated the wound [19].

Therefore, we independently developed a CSD by combining the characteristics of the PVA sponge [5]. The principle of hemostasis was similar to XStat [20]. However, through practice, we found that the original device could not completely inject the sponge into deep wounds due to the short injection tube. Thus, we improved the device by incorporating a reverse push-type syringe structure to solve this problem. This study showed that the MCSD offered the following advantages.

### Rapid hemostatic effect

We found that the use of the MCSD was associated with less post-treatment blood loss, less hemostasis time, more stable vital signs like MAP, more stable hemoglobin level,and a higher success rate than SG. In addition to the rapid hemostatic effect of the PVA sponge in MCSD, the unique structural characteristics of MCSD also played a huge role in conferring these advantages. The MCSD adopts a reverse push-type syringe structure to ensure that the series of PVA sponges can enter the deep wound smoothly. In comparison with gauze, the MCSD, which has a diameter of only 10 mm, id more likely to enter narrow and complex wounds caused by bullets or explosive fragments.

### Stable hemostatic effect

Lander et al. showed that limb movement after the application of hemostatic materials can lead to rebleeding [21]. Although the same experiment was not performed for this study, the MCSD group showed no further bleeding after successful hemostasis despite the process of handling. We considered that this could be attributed to the close compression of the PVA sponge after expanding according to the CT scan results. Moreover, the shape advantages of the prism after expansion made it easier for the dressing to remain stuck and not fall off.

### Improved removal efficiency

A retrospective study by Warina et al. reported retention of hemostatic material in the wound when XStat was used to treat penetrating wounds [19]. However, no retention was observed in the MCSD group in the present study because of the chain structure of MCSD. Moreover, no significant difference in the removal time was observed between the MCSD and gauze groups. We considered that this may reduce the possibility of wound enlargement by residual material and obviate the need for a secondary extraction.

### Good biocompatibility

Medical-grade PVA sponge is widely used in vacuum suction dressings, implantable medical devices, and other medical fields. In this study, TEM and HE staining results showed that the application of MCSD did not lead to significant necrosis of the femoral artery and muscle tissue.

### Operability and portability

The MCSD can be used single-handed at any time to meet the needs of soldiers for self-rescue and mutual rescue. The compressed PVA sponge can absorb blood and expand, exerting pressure on the surrounding area. We did not even pressurize the wound after injecting the PVA sponge in the experiment. Moreover, the MCSD is sufficiently portable, with a single MCSD weighing only 15 g.

In summary, these findings indicate that the MCSD meets the requirements of war trauma emergency hemostatic dressings, showing a good hemostatic effect, short hemostasis time, good safety performance, and short removal time [22].

Nevertheless, this study also had some limitations. First, although the device is designed to control bleeding at all junctional sites, due to the limitations imposed by the experimental conditions, only the groin region was tested. Further experiments are needed to evaluate hemostasis in different anatomical locations such as the buttock, armpit, and neck. Next, Wang et al. chose to shoot the pistol at close range 3 m away and missed the femoral artery in 36 animals (success rate, 83%) [13]. Similar to the anatomy of humans, the femoral veins and arteries are next to each other in Bama miniature pigs. Therefore, in bleeding models based on femoral artery injury, injury to the femoral vein during establishment of the groin bullet wound model is an unavoidable problem.

Kheirabadi et al. applied a tourniquet in a porcine femoral artery injury bleeding model, and repaired the injured femoral artery and sutured it layer-by-layer to observe complications for 1 week after hemostasis [23]. In the present experiment, the Bama miniature pig model with a bullet penetrating the artery in the inguinal region was more difficult to repair than a simple femoral artery injury model. The bullet also caused comminuted fractures of the pelvis and femur, which affected subsequent functional recovery. However, since the observation time for this study was limited to 3 h, no further damage control surgery was performed.

Furthermore, the XStat, junction tourniquet, etc. have been applied to patients in pre-hospital trauma and bleeding, highlighting the need to apply the MCSD in clinical practice. Lastly, study showed that use of local pro-coagulant hemostatic agents could improve the hemostatic effect [24]. In future studies, we aim to further improve the MCSD by designing different types of injection tubes and evaluate the improved MCSDs in animal explosion models and different anatomical locations such as the buttock, armpit, and neck.

## Summary

The MCSD met the battlefield’s requirements of speedy hemostasis and biosafety when applied for junctional hemorrhage in Bama miniature pigs. Moreover, in comparison with the conventional hemostatic technique, it showed more stable performance for deep wound hemostasis. The experiment provides a theoretical and experimental basis for the application of the MCSD in the treatment of hemorrhage in the battlefield in the future.

## Data Availability

The datasets used and analyzed during the current study are available from the corresponding author on reasonable request.

## Abbreviations

MCSD: Modified chain-based sponge dressin
SG: standard medical gauze
PVA: polyvinyl alcohol
CoTCCC: Committee on Tactical Combat Casualty
CRoC: Combat Ready Clamp
JETT: Junctional Emergency Treatment Tool
SJT: SAM Junctional Tourniquet
MAP: Mean Aarterial Pressure
SEM: scanning electron microscope
HG: Hemoglobin
Plt: blood platelet
PT: prothrombin time
APTT: activated partial thromboplastin time
